# Temporal dynamics of hemispheric interactions in visual word recognition using a repetition priming paradigm: modulation by stimulus onset asynchrony

**DOI:** 10.3389/fpsyg.2025.1553708

**Published:** 2025-08-01

**Authors:** Sangyub Kim, Kichun Nam

**Affiliations:** ^1^Department of Psychology, Chonnam National University, Gwangju, Republic of Korea; ^2^School of Psychology, Korea University, Seoul, Republic of Korea

**Keywords:** interhemispheric interaction, visual word recognition, stimulus onset asynchrony (SOA), hemispheric specialization, lexical decision task (LDT)

## Abstract

**Introduction:**

Understanding how the two cerebral hemispheres interact during visual word recognition is central to describing the temporal and spatial dynamics of language processing. This study examined the role of stimulus onset asynchrony (SOA) in modulating interhemispheric interactions during a primed-lateralized lexical decision task. We focused on how the timing between prime and target presentations influences the contributions of the left and right hemispheres, using word familiarity.

**Method:**

Participants performed a lexical decision task involving lateralized prime-target word pairs. Stimuli were presented either to the left visual field (LVF) or the right visual field (RVF) across three SOA conditions: 0 ms (simultaneous), 100 ms, and 200 ms (sequential). Word familiarity served as an indicator of lexical proficiency to assess hemispheric involvement across different temporal intervals. Interhemispheric interaction was inferred from performance patterns across visual field pairings and SOAs.

**Results:**

At a 100 ms SOA, when primes were presented to the LVF and targets to the RVF, response patterns indicated dominant involvement of the right hemisphere's visual-perceptual system in initiating interhemispheric processing. In contrast, at a 200 ms SOA, when primes were shown to the RVF and targets to the LVF, results reflected a stronger influence of the left hemisphere's lexical-semantic processing mechanisms in modulating interhemispheric effects.

**Discussion:**

These findings underscore the critical role of temporal dynamics in shaping hemispheric contributions to visual word recognition. The differential effects observed at 100 ms and 200 ms SOAs suggest that interhemispheric integration is not static but time-sensitive, with each hemisphere showing varying degrees of influence depending on the temporal sequencing of activation. The results imply that lexical processing is contingent not only on visual field presentation but also on the precise timing of hemispheric engagement.

## Introduction

The two cerebral hemispheres engage in continuous interaction via the corpus callosum, a major white-matter track that enables bidirectional signal transfer within millisecond time frames in the intact brain (Aboitiz and Montiel, 2003; Hofer and Frahm, 2006). Rapid interhemispheric dynamics underlie high-level cognitive functions such as language processing, which depend on the swift integration of information across hemispheres (Gazzaniga, 2000; Hofer and Frahm, 2006). Visual word processing, a critical component of reading fluency, offers a valuable window into these hemispheric interactions (Banich, 2003). Investigating how visual words are processed by each hemisphere provides clues to understand the neural mechanisms that support reading.

An intriguing approach to examining hemispheric dynamics is the visual half-field presentation paradigm (Kim et al., 2020, 2022a,b; Kim and Nam, 2023a,b,c; Kim et al., 2023a, 2024a,b). This method involves briefly presenting a visual stimulus in the parafoveal field, ~2–5 degrees of visual angle from the center of gaze, to prevent direct fixation. By projecting the stimulus to the parafoveal field, the paradigm ensures initial activation of the contralateral hemisphere corresponding to the opposite visual field (Bourne, 2006). For instance, a stimulus presented in the left parafoveal field activates the right hemisphere (RH), while one presented in the right parafoveal field activates the left hemisphere (LH). This paradigm is particularly effective for exploring interhemispheric interactions through the sequential presentation of stimuli in alternating parafoveal fields with precisely controlled time intervals. For example, presenting a stimulus in the left parafoveal field before a stimulus in the right parafoveal field enables researchers to examine the influence of RH processing on subsequent LH processing.

Previous studies have employed the visual half-field presentation paradigm to investigate interhemispheric interactions in visual word recognition, with particular focus on word familiarity as a measure of processing proficiency. We prioritized familiarity over frequency as a key lexical variable, given its greater relevance to the cognitive mechanisms underlying visual word recognition. While frequency refers to the objective occurrence of a word within a linguistic corpus, familiarity captures not only frequency but also the subjective ease with which a word is recognized. This dual nature of familiarity integrates both exposure and cognitive accessibility, offering a more comprehensive measure of lexical proficiency than frequency alone. Kim et al. (2022a) utilized the visual half field presentation paradigm by presenting stimuli with different familiarity levels in unilateral and bilateral parafoveal fields to assess bilateral redundancy gain (BRG), an index of interhemispheric facilitation calculated as the difference in reaction times (RTs) between unilateral and bilateral presentations. Their findings revealed a significant BRG for words with the highest familiarity level, in contrast to the other three levels of familiarity (least familiar, moderately unfamiliar, and moderately familiar). This result indicates more facilitative interhemispheric interactions during the visual recognition of highly familiar words compared to less familiar words. Further supporting this, Kim et al. (2023a) examined electrophysiological responses to words across the four levels of familiarity, applying a Granger causality analysis to determine the directional interaction between hemispheres. They observed stronger asymmetric interactions from the RH to the LH for words with the highest familiarity level compared to those with the least familiarity.

Although previous studies have demonstrated that interhemispheric interactions in visual word recognition are modulated by word familiarity—a phenomenon known as the familiarity effect, whereby familiar words are recognized more rapidly and/or accurately than unfamiliar ones (Kim et al., 2022a, 2023a)—these investigations did not address alterations in interhemispheric interactions occurring within brief temporal intervals. To date, the only study to examine such time-sensitive changes in interhemispheric interactions influenced by word familiarity is Kim and Nam (2024). In their work, Kim and Nam (2024) employed a primed-lateralized lexical decision task with a 100-ms stimulus onset asynchrony (SOA) between the presentation of identical primes and targets to explore interhemispheric dynamics in visual word recognition. They evaluated the effects of word familiarity under conditions where primes and targets were presented sequentially in either the same or opposite visual fields with the 100-ms SOA. Their findings revealed a significant interhemispheric word familiarity effect when primes were presented in the left visual field (LVF)/RH and targets in the right visual field (RVF)/LH. This result indicates a facilitative interaction from RH processing to LH processing within the 100-ms interval, aligning with the electrophysiological findings of Kim et al. (2023a).

As demonstrated by Kim and Nam (2024), within a 100-ms SOA, the presentation of a prime in the LVF initially activates the RH, significantly facilitating subsequent processing of a target presented in the RVF, which projects to the LH. This facilitative effect may be attributed to the visual-perceptual specialization of RH processing, which is closely linked to word familiarity processing (Pollatsek et al., 1975; Yang and Beck, 2023). The RH's visual-perceptual system plays a crucial role in encoding and interpreting the physical attributes of words, such as letter shapes, spacing, and word length. These attributes directly interact with an individual's lexical knowledge, including familiarity, thereby enhancing the analytical lexical processing of the LH. However, the familiarity effect from RH to LH is likely modulated by the temporal dynamics of hemispheric activation. In this context, Hauk et al. (2006) investigated the time course of access to psycholinguistic properties during visual word recognition by analyzing electrophysiological responses in a lexical decision task. Their regression analysis of EEG data revealed an early influence of word length and letter n-gram frequency (e.g., bigram or trigram frequencies) at ~90 ms. Lexical frequency effects—whereby high-frequency words are recognized more rapidly and accurately than low-frequency counterparts—emerged at ~110 ms post-stimulus. In contrast, semantic coherence, reflecting the consistency of meaning across morphologically related word forms, showed significant correlations with electrophysiological responses around 160 ms. Notably, this timing aligned with the onset of the lexicality effect, meaning temporally distinct stages of psycholinguistic processing during visual word recognition.

In the present study, we manipulated the SOA between the prime and target to intervals of 100 and 200 ms, following the framework proposed by Holcomb and Grainger (2007). Their bimodal interactive model delineates three stages of word recognition: visual feature processing, prelexical processing, and lexical-level representation. According to this model, visual feature processing occurs within the first ~100 ms post-stimulus presentation, transitioning to prelexical and lexical stages thereafter. Consequently, sequential presentation in the left and right parafoveal visual fields, combined with these distinct SOAs, is expected to produce different interhemispheric interaction patterns. These differences arise due to the specialized roles of each hemisphere in visual word processing. Hemispheric specialization, as supported by previous research (Barca et al., 2011; Binder et al., 2009; Brysbaert, 2004; Chu et al., 2020; Hellige, 1996; Van der Haegen et al., 2009), posits that the RH primarily engages in global visual-perceptual processing, whereas the LH predominantly undertakes lexical processing. Based on these functional distinctions, we hypothesized that a significant interhemispheric interaction from the RH to LH would emerge at an SOA of 100 ms, replicating the findings of Kim and Nam (2024). This interaction would reflect the RH's dominance in early visual-perceptual processing (Hellige, 1996; Lamb and Robertson, 1988; Lamb et al., 1989, 1990). Conversely, at an SOA of 200 ms, significant interhemispheric interaction from the LH to RH was anticipated, aligning with the temporal sequence in which lexical processing in the LH succeeds earlier visual-perceptual processing (Mohr et al., 1994; Binder et al., 2009).

### The current study

In this study, we aimed to explore alterations in interhemispheric interactions during the development of reading proficiency, using word familiarity as an indicative parameter. Specifically, we investigated how variations in the SOA between identical prime and target presentations influence interhemispheric interactions during visual word processing, with a particular focus on the familiarity effect. Given that interhemispheric transfer typically occurs within ~50 ms (Korkmaz et al., 2022), we hypothesized that extending the SOA from 100 to 200 ms would yield differential effects on interhemispheric interactions in visual word processing. The decision to include the 200 ms SOA condition was grounded in the possibility that this extended interval would enable a more pronounced familiarity effect, even when the prime was presented in the RVF/LH. This expectation is based on the sequential nature of visual word recognition, where lexical processing in the LH follows visual-perceptual processing in the RH (Hauk et al., 2006). Therefore, we employed two SOA conditions −100 and 200 ms—to examine their effects on interhemispheric interactions during visual word processing. We hypothesized that a significant familiarity effect would be observed in the 100 ms SOA condition when the prime was presented in the LVF/RH. This is consistent with the RH's role in visual-perceptual processing during the early stages of word recognition (Hauk et al., 2006). In contrast, we anticipated a significant familiarity effect in the 200 ms SOA condition when the prime was presented in the RVF/, where lexical processing could more fully unfold. This extended SOA would provide additional time for lexical processing, potentially amplifying the familiarity effect when the LH is primed.

The investigation of the SOA between the prime and target in interhemispheric interactions also requires consideration of the 0 ms SOA condition. If, as hypothesized, SOA influences interhemispheric interactions, the 0 ms SOA condition should exhibit distinct interaction patterns compared to the 100 and 200 ms SOA conditions. In this study, the 0 ms SOA condition refers to the simultaneous presentation of the prime and target, with both stimuli appearing concurrently in the left and right parafoveal visual fields, thereby engaging both hemispheres. This bilateral activation is prompted by two types of visual presentation: bilateral visual field (BVF) presentation, in which stimuli are simultaneously presented in the left and right parafoveal fields, and central visual field (CVF) presentation, which activates both hemispheres via foveal vision. These two modalities were used as control conditions. The aim was to assess the impact of SOA in each experimental condition by comparing responses in the 0 ms condition with those observed in the 100 and 200 ms conditions.

The impact of SOA was quantified by subtracting behavioral responses (reaction times and accuracy) at the 100 and 200 ms SOA conditions from those at 0 ms SOA condition. We hypothesized that if SOA indeed modulates interhemispheric dynamics in familiarity-based visual word recognition, the familiarity effect observed at longer SOAs would be diminished or abolished relative to the 0 ms condition. This attenuation would suggest that the robust familiarity effects evident in the BVF and CVF control conditions—where both hemispheres contribute simultaneously—are weakened under sequential prime-target presentation. This interpretation is grounded in the notion that in the control conditions, both hemispheres successfully interact for word familiarity processing, thereby balancing or nullifying any differences observed in the sequential presentations.

## Method

### Participants

A total of 48 participants were initially recruited from Korea University. However, six participants were excluded from the final analysis due to their handedness (five left-handed and one ambidextrous), as assessed by the Edinburgh Handedness Inventory (*M* = 8.52, *SD* = 1.47). As a result, the final sample consisted of 42 right-handed participants (26 males and 16 females), with a mean age of 27.14 years (*SD* = 4.58). The study was conducted in accordance with the ethical principles delineated in the 1964 Declaration of Helsinki, and approved by the Institutional Review Board of Korea University (KUIRB-2018-0086-01). All participants were informed of the study's ethical guidelines and provided written informed consent prior to participation. Participants were compensated with a payment of 7,000 Korean won for their involvement. All participants reported normal or corrected-to-normal vision.

### Experimental task and procedure

A primed-lateralized lexical decision task was utilized to probe the interhemispheric processing of identical prime and target stimuli, contingent on two distinct SOAs of 100 and 200 ms. Participants were first acquainted with the task instructions and subsequently commenced the task by pressing the space bar on the keyboard. A central fixation point was displayed for 2,000 ms to secure fixation, followed by a brief 50 ms presentation of the prime in either the LVF or RVF, thereby initiating propagation to the contralateral hemisphere. A mask (“X#@X#@”) was concomitantly displayed in the opposing visual field to that of the prime. Subsequently, an empty screen was presented for either 50 or 150 ms, allowing time for interaction with the target, which was then displayed in the parafoveal visual field for 180 ms, accompanied by the mask. Participants were instructed to respond as quickly and accurately as possible within a 2,000-ms time window, indicating whether the presented stimulus was a word or a non-word.

### Materials

In the present study, we utilized the same materials as those used in Kim and Nam (2024) to directly compare their findings regarding the interhemispheric familiarity effect. The items in Kim and Nam (2024) were selected in a non-biased manner, adhering to a predetermined distribution from diverse sources: newspapers (20%), movies (10%), published papers/articles (30%), and internet blogs (40%), following the proportional guidelines established by the Korean Sejong Corpus (Kang and Kim, 2009). The objective was to examine the visual processing of words encountered in everyday contexts. Lexical items with noun-verb ambiguities were excluded, as the study focused primarily on noun processing.

For the evaluation of word familiarity, Kim et al. (2020) employed a Likert scale, where participants rated their familiarity with each word on a scale from 1 (least familiar) to 7 (most familiar), following the methodology used in Kreuz (1987). A total of 300 Korean words were assessed and were incorporated into the present study. The words were classified into four levels of subjective familiarity: F1 (least familiar; e.g., distribution), F2 (moderately unfamiliar; e.g., phenomenon), F3 (moderately familiar; e.g., passion), and F4 (most familiar; e.g., tomorrow). Each level comprised 75 words, resulting in a total of 300 items.

Additionally, we controlled for semantic properties (number of objective meanings), physical length (number of strokes, phonemes, syllables, and morphemes), and frequency (log-transformed first-syllable frequency) across all familiarity levels (Table 1). The average familiarity ratings and standard errors for each level were as follows: F1: 4.21 ± 0.08, F2: 4.61 ± 0.10, F3: 5.10 ± 0.09, and F4: 5.46 ± 0.09. A significant difference in familiarity was observed across the levels [*F*_(3, 296)_ = 35.415, *p* < 0.001, $\eta_{p}^{2}$ = 0.264], and *post-hoc* Bonferroni tests confirmed significant differences between each pair of familiarity levels. Thus, 300 Korean words and 300 non-word stimuli were employed in this study to maintain a balanced response for the lateralized lexical decision task. Non-word stimuli were constructed using Korean syllables, including both syllables that conformed to orthographic and phonological rules but lacked semantic meaning (i.e., 눕판이), and those that violated these linguistic conventions and were also meaningless (i.e., 븆책에). All non-words were confirmed to be absent from the Sejong Corpus, ensuring that they possessed no lexical status in Korean. To minimize potential confounding effects from string length, the syllable length of non-words was exactly matched to that of real words, thereby controlling for length-related influences on visual word recognition (Kim et al., 2022b).

**Table 1:** Summary information for matched lexical variables across familiarity levels (F1, F2, F3, F4): Mean values presented with standard deviations in parentheses.

**Lexical variables**	**Level of familiarity**			
	**F1**	**F2**	**F3**	**F4**
Strokes	19.43 (4.92)	18.96 (4.46)	19.19 (4.10)	17.76 (4.53)
Phonemes	8.21 (1.51)	8.05 (1.64)	8.09 (1.31)	7.89 (1.74)
Syllables	3.29 (0.49)	3.24 (0.57)	3.19 (0.46)	3.09 (0.60)
Morphemes	2.10 (0.34)	2.08 (0.30)	2.12 (0.33)	2.09 (0.29)
Objective meanings	1.35 (0.69)	1.65 (1.80)	1.49 (1.07)	1.51 (1.10)
(log) First syllable frequency	3.76 (0.48)	3.68 (0.58)	3.75 (0.41)	3.73 (0.47)
Subjective familiarity	4.21 (0.74)	4.61 (0.91)	5.10 (0.75)	5.46 (0.78)

### Experimental condition

Experiment was structured around several factors, each with distinct levels. The visual field (VF) factor, comprising two levels (RVF-LVF/LVF-RVF), denoted the sequential visual fields for prime and target presentation. For instance, RVF-LVF signifies that the prime was presented in the RVF/LH, and the target in the LVF/RH. This factor was designed to examine the interhemispheric interaction by presenting stimuli in opposing visual fields. The SOA condition, with levels of 100 and 200 ms, was implemented to investigate the influence of temporal separation between prime and target on interhemispheric interaction during visual word processing. The familiarity factor, encompassing four levels (F1/F2/F3/F4), aimed to explore the interhemispheric interaction as modulated by word familiarity. Furthermore, a lexicality factor was introduced to discern the differential responses to word and non-word stimuli, reflecting the presence or absence of processing experience. This multifaceted design facilitated an investigation into the interhemispheric processing dynamics associated with word familiarity. In addition to these experimental factors, control factor was introduced, employing two levels of BVF and CVF, referred to as the SOA 0 ms. BVF denotes the simultaneous presentation of stimuli in both left and right parafoveal fields, whereas CVF refers to central presentation within the foveal region. These control levels were utilized to calculate the SOA effect for the aforementioned experimental factors. The SOA effect was quantified by subtracting the responses at 100 and 200 ms SOAs from those at 0 ms (BVF and CVF), thereby providing a rigorous basis for assessing the impact of SOA on interhemispheric interactions.

### Apparatus

Participants were positioned with their chin resting on a chinrest, maintaining a distance of 65 cm between the nasion and the monitor. This fixed position was maintained throughout the experimental task to minimize variability. Stimuli were presented on an LG monitor with the capability to display RGB colors. Participants' responses were captured via a keyboard strategically positioned in front of them. Specific instructions were provided for response mechanisms: participants were directed to use their right index finger to press the “slash” button for word responses, and their left index finger to press the “z” button for non-word responses. The assignment of response hand was counterbalanced across participants to mitigate potential biases. The entire experimental procedure was conducted using Experimental Psychology Software (E-prime), a specialized tool chosen for its precision and reliability in psychological research.

## Results

Data were collected for RTs and ACC in the primed-lateralized lexical decision task. Notably, ACC for words and non-words for all participants fell within three standard deviations, with the exception of one participant. This necessitated the exclusion of data from that individual in the final analysis.

### Familiarity effect on the interhemispheric interactions in RTs and ACC

**Figure 1:**
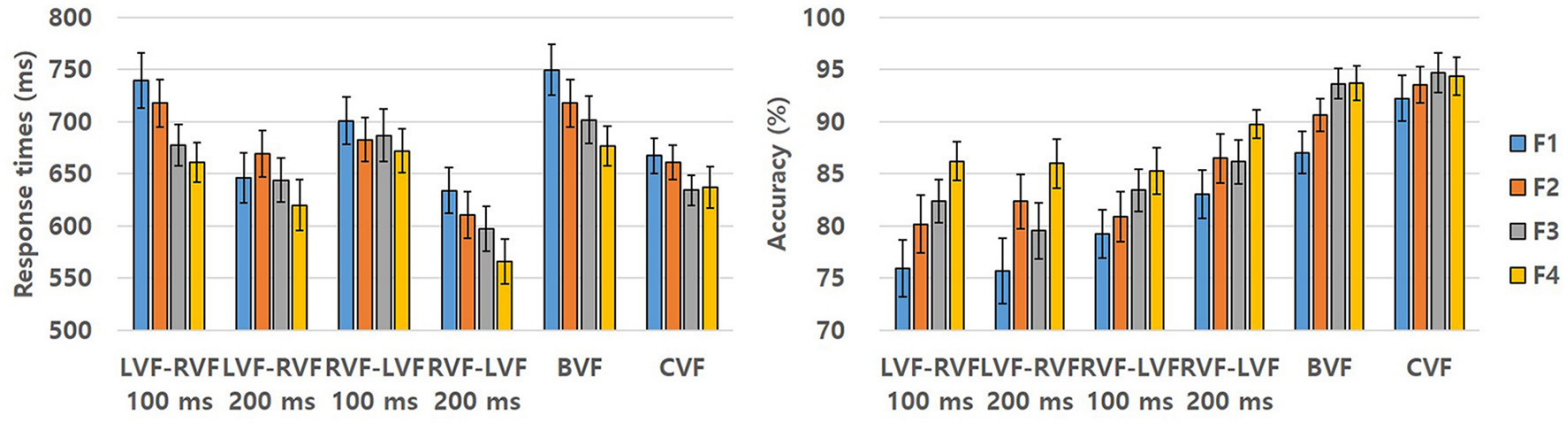
Graphical representation illustrating the collective behavioral responses of words delineated by RTs and ACC within each experimental condition, pertaining to the familiarity effect in Experiment. The bars are accompanied by lines indicating standard errors. F1, The most unfamiliar level; F2, Slightly unfamiliar level; F3, Slightly familiar level; F4, The most familiar level.

Comprehensive behavioral responses for the familiarity effect, including both RTs and ACC, are detailed in Table 2 and further illustrated in Figure 1. A mixed-effect regression analysis was utilized to investigate the effects of familiarity on RTs and ACC for words. The model incorporated fixed effects, including familiarity (F1, F2, F3, F4), visual field (VF) with two levels (LVF-RVF, RVF-LVF), SOA with two levels (100, 200 ms), and their two-way interactions (familiarity × VF, familiarity × SOA, VF × SOA), along with a three-way interaction (familiarity × VF × SOA). Random effects were also included to account for variability among participants and items.

**Table 2:** Comprehensive summary of the behavioral responses observed in terms of RTs (ms) and ACC (%) within each experimental condition for the interhemispheric familiarity effect.

**VF**	**SOA (ms)**	**Level of familiarity**							
		**F1**		**F2**		**F3**		**F4**	
		**RTs**	**ACC**	**RTs**	**ACC**	**RTs**	**ACC**	**RTs**	**ACC**
LVF-RVF	100	739 (169)	75.9 (17.7)	718 (146)	80.2 (17.8)	677 (126)	82.4 (13.2)	661 (122)	86.2 (11.9)
	200	646 (156)	75.7 (20.0)	669 (144)	82.3 (16.8)	644 (133)	79.5 (17.2)	620 (154)	86.0 (15.0)
RVF-LVF	100	701 (145)	79.2 (14.9)	683 (135)	80.9 (15.3)	687 (163)	83.4 (13.0)	672 (135)	85.3 (14.3)
	200	634 (140)	83.1 (14.9)	610 (143)	86.5 (15.0)	597 (139)	86.2 (13.4)	566 (138)	89.7 (8.7)
BVF	0	750 (157)	87.0 (12.8)	718 (146)	90.6 (9.9)	702 (145)	93.6 (9.3)	676 (122)	93.7 (10.6)
CVF	0	667 (109)	92.2 (13.9)	661 (107)	93.5 (11.0)	634 (92)	94.7 (12.2)	637 (126)	94.4 (11.7)

The values within brackets represent the corresponding standard deviations.

The initial analysis, conducted using the lmer function in R, centered on RTs. The results showed significant main effects of familiarity [β = −8.412, *SE* = 2.574, *t* = −3.268, *p* = 0.001], visual field [VF; β = −14.069, *SE* = 2.752, *t* = −5.112, *p* < 0.001], and SOA [β = −34.808, *SE* = 2.746, *t* = −12.677, *p* < 0.001]. A significant two-way interaction effect was observed between VF and SOA [β = −8.332, *SE* = 2.747, *t* = −3.003, *p* = 0.002], while the two-way interaction effects between familiarity and VF and between familiarity and SOA were not significant [β = −0.531, *SE* = 1.742, *t* = −0.305, *p* = 0.760 for familiarity × VF; β = 0.992, *SE* = 1.739, *t* = 0.571, *p* = 0.568 for familiarity × SOA]. A significant three-way interaction effect emerged among all factors [β = −3.642, *SE* = 1.743, *t* = −2.090, *p* = 0.037]. The main effect of familiarity indicated more rapid RTs for familiar words. Additionally, the main effect of VF revealed that the sequence RVF-LVF yielded faster responses than LVF-RVF. The main effect of SOA demonstrated quicker responses for SOA 200 ms compared to SOA 100 ms. Furthermore, the significant two-way interaction between VF and SOA revealed faster responses for SOA 200 ms compared to SOA 100 ms in both LVF-RVF and RVF-LVF sequences [β = −21.180, *SE* = 2.474, *t* = −8.560, *p* < 0.001 for LVF-RVF; β = −43.190, *SE* = 3.875, *t* = −11.200, *p* < 0.001 for RVF-LVF]. The significant three-way interaction among all factors indicated that the familiarity effect was pronounced when the prime and target were sequentially presented at LVF-RVF with SOA 100 ms [β = −13.190, *SE* = 4.274, *t* = −3.056, *p* = 0.002] and RVF-LVF with SOA 200 ms [β = −11.591, *SE* = 4.191, *t* = −2.766, *p* = 0.006]. Conversely, no significant familiarity effect was observed when the prime and target were sequentially presented at LVF-RVF with SOA 200 ms [β = −3.372, *SE* = 4.110, *t* = −0.821, *p* = 0.413] or RVF-LVF with SOA 100 ms [β = −6.389, *SE* = 4.040, *t* = −1.581, *p* = 0.115].

The analysis of ACC was conducted using the glmer function in R, revealing significant main effects of familiarity [β = 0.126, *SE* = 0.036, *z* = 3.512, *p* < 0.001], visual field [VF; β = 0.142, *SE* = 0.032, *z* = 4.451, *p* < 0.001], and SOA [β = 0.085, *SE* = 0.032, *z* = 2.659, *p* = 0.008]. A significant two-way interaction effect was observed between VF and SOA [β = 0.102, *SE* = 0.032, *z* = 3.202, *p* = 0.001], while the two-way interaction effects between familiarity and VF and between familiarity and SOA were not significant [β = −0.027, *SE* = 0.020, *z* = −1.350, *p* = 0.177 for familiarity × VF; β = 0.018, *SE* = 0.020, *z* = 0.880, *p* = 0.379 for familiarity × SOA]. The three-way interaction effect among all factors was not significant [β = 0.003, *SE* = 0.020, *z* = 0.130, *p* = 0.897]. The main effect of familiarity indicated greater ACC for familiar words. Additionally, the main effect of VF revealed higher ACC when the prime and target were sequentially presented at RVF-LVF compared to LVF-RVF. The main effect of SOA demonstrated increased ACC for SOA 200 ms compared to SOA 100 ms. Furthermore, the significant two-way interaction between VF and SOA revealed that SOA 200 ms yielded higher ACC than SOA 100 ms when the prime and target were sequentially presented at RVF-LVF [β = 0.192, *SE* = 0.047, *z* = 4.059, *p* < 0.001], while no ACC difference was observed between SOA 100 and 200 ms when the prime and target were sequentially presented at LVF-RVF [β = −0.012, *SE* = 0.043, *z* = −0.272, *p* = 0.786].

### Lexicality effect on the interhemispheric interactions in RTs and ACC

**Figure 2:**
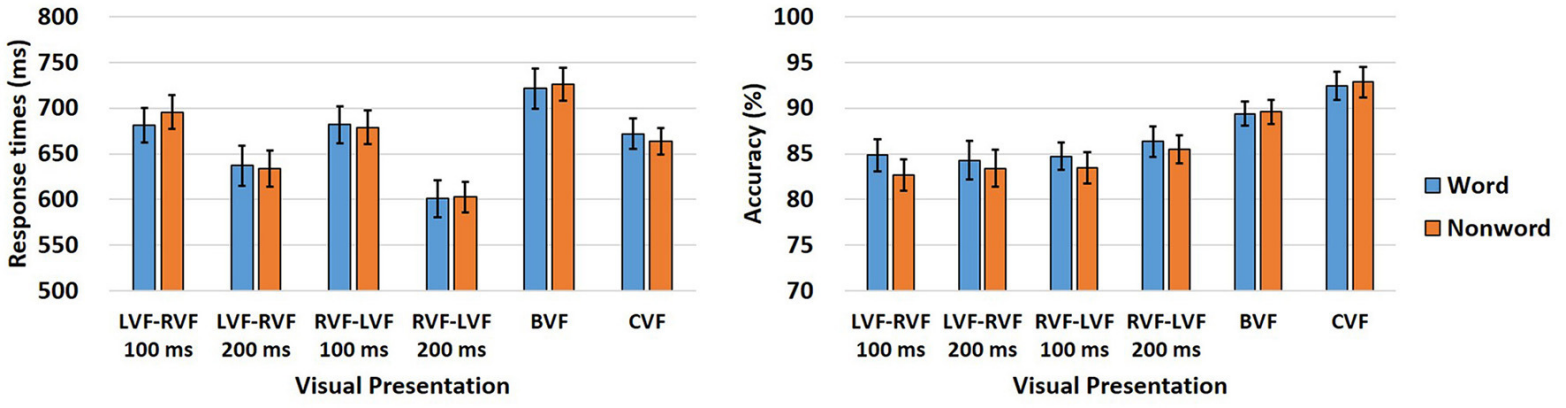
Graphical depiction illustrating the amalgamated behavioral responses as represented by RTs and ACC within each experimental condition, regarding the lexicality effect in Experiment. The bars are accompanied by lines denoting standard errors.

Detailed within Table 3 are the comprehensive behavioral responses pertaining to the lexicality effect, encompassing both RTs and ACC. Further visual representation of these findings can be found in Figure 2. A mixed-effect regression analysis was conducted to investigate the effect of lexicality on RTs and ACC for both words and non-words. The model incorporated fixed effects, including lexicality (categorized as word or non-word), visual field (VF; denoted as LVF-RVF or RVF-LVF), SOA (defined as 100 or 200 ms), and their respective two-way interactions (lexicality × VF, lexicality × SOA, VF × SOA), along with a three-way interaction (lexicality × VF × SOA). Random effects were also included in the model to account for variability across participants and items.

**Table 3:** Comprehensive summary of the behavioral responses observed in terms of RTs (ms) and ACC (%) within each experimental condition for the lexicality effect.

**VF**	**SOA (ms)**	**Word**		**Non-word**	
		**RTs**	**ACC**	**RTs**	**ACC**
LVF-RVF	100	681 (122)	84.8 (11.2)	696 (117)	82.7 (11.0)
	200	637 (140)	84.3 (13.4)	634 (128)	83.4 (12.7)
RVF-LVF	100	682 (128)	84.7 (9.5)	679 (117)	83.5 (11.0)
	200	601 (131)	86.3 (10.7)	603 (106)	85.5 (9.8)
BVF	0	721 (140)	89.4 (8.4)	726 (117)	89.6 (8.4)
CVF	0	672 (106)	92.4 (9.7)	663 (92)	92.8 (10.7)

The values within brackets represent the corresponding standard deviations.

The initial analysis, conducted using the lmer function in R, centered on RTs. The results unveiled significant main effects of lexicality [β = −5.894, *SE* = 2.947, *t* = −2.000, *p* = 0.046], visual field [VF; β = −9.840, *SE* = 1.938, *t* = −5.077, *p* < 0.001], and SOA [β = −33.534, *SE* = 1.936, *t* = −17.322, *p* < 0.001]. Noteworthy two-way interaction effects emerged between lexicality and VF [β = 4.152, *SE* = 1.937, *t* = 2.143, *p* = 0.032], and between VF and SOA [β = −6.039, *SE* = 1.937, *t* = −3.118, *p* = 0.002], while the interaction between lexicality and SOA was not significant [β = 1.194, *SE* = 1.936, *t* = 0.617, *p* = 0.537]. Furthermore, the three-way interaction among all factors was not significant [β = 2.642, *SE* = 1.936, *t* = 1.364, *p* = 0.173]. The significant main effect of lexicality revealed quicker RTs for words compared to non-words. The main effect of VF indicated more rapid responses when the prime and target were sequentially presented at the RVF and LVF, respectively. The main effect of SOA demonstrated swifter responses for SOA 200 ms compared to SOA 100 ms. Additionally, the two-way interaction between lexicality and VF revealed a significant lexicality effect when the prime and target were sequentially presented at the LVF and RVF [β = −10.005, *SE* = 3.568, *t* = −2.804, *p* = 0.005], meaning faster RTs for non-words than for words in the LVF-RVF presentation, but not when sequentially presented at the RVF and LVF [β = −1.156, *SE* = 3.643, *t* = −0.317, *p* = 0.751]. The interaction between VF and SOA indicated faster responses for SOA 200 ms compared to SOA 100 ms in both LVF-RVF and RVF-LVF prime and target presentations [β = −27.296, *SE* = 2.706, *t* = −10.090, *p* < 0.001 for LVF-RVF; β = −39.401, *SE* = 2.743, *t* = −14.360, *p* < 0.001 for RVF-LVF].

Following the analysis of RTs, ACC was examined utilizing the glmer function in R. The findings revealed significant main effects of lexicality [β = 0.177, *SE* = 0.045, *z* = 3.946, *p* < 0.001] and SOA [β = −0.046, *SE* = 0.023, *z* = 1.969, *p* = 0.049], though the main effect of visual field (VF) was not significant [β = 0.044, *SE* = 0.023, *z* = 3.946, *p* < 0.001]. A noteworthy two-way interaction effect between lexicality and VF emerged as significant [β = −0.100, *SE* = 0.023, *z* = −4.255, *p* < 0.001], while the interaction effects between lexicality and SOA, and between VF and SOA were not significant [β = −0.036, *SE* = 0.023, *z* = −1.522, *p* = 0.128 for lexicality × SOA; β = 0.043, *SE* = 0.023, *z* = 1.854, *p* = 0.064 for VF × SOA]. Moreover, a significant three-way interaction among all factors was observed [β = −0.059, *SE* = 0.023, *z* = −2.502, *p* = 0.012]. The main effect of lexicality indicated greater ACC for words compared to non-words, and the main effect of SOA revealed higher ACC for SOA 200 ms compared to SOA 100 ms. Furthermore, the two-way interaction between lexicality and VF showed a significant simple main effect of lexicality, meaning higher ACC for non-words than for words, when the prime and target were sequentially presented at the LVF and RVF [β = 0.259, *SE* = 0.048, *z* = 5.345, *p* < 0.001], but not when presented at the RVF and LVF [β = 0.079, *SE* = 0.055, *z* = 1.433, *p* = 0.152]. The three-way interaction among all factors indicated a significant simple main effect of lexicality, meaning higher ACC for non-words than words, in both SOA 100 and 200 ms for LVF-RVF prime and target presentation [β = 0.234, *SE* = 0.058, *z* = 4.045, *p* < 0.001 for SOA 100 ms; β = 0.279, *SE* = 0.058, *z* = 4.839, *p* < 0.001 for SOA 200 ms]. Conversely, the simple main effect of lexicality was only significant in SOA 100 ms, not in SOA 200 ms, when the prime and target were sequentially presented at the RVF and LVF [β = 0.158, *SE* = 0.064, *z* = 2.46, *p* = 0.014 for SOA 100 ms; β = −0.037, *SE* = 0.063, *z* = −0.581, *p* = 0.561 for SOA 200 ms].

### Impact of SOA 0 ms on interhemispheric interactions assessed through BVF performance

**Figure 3:**
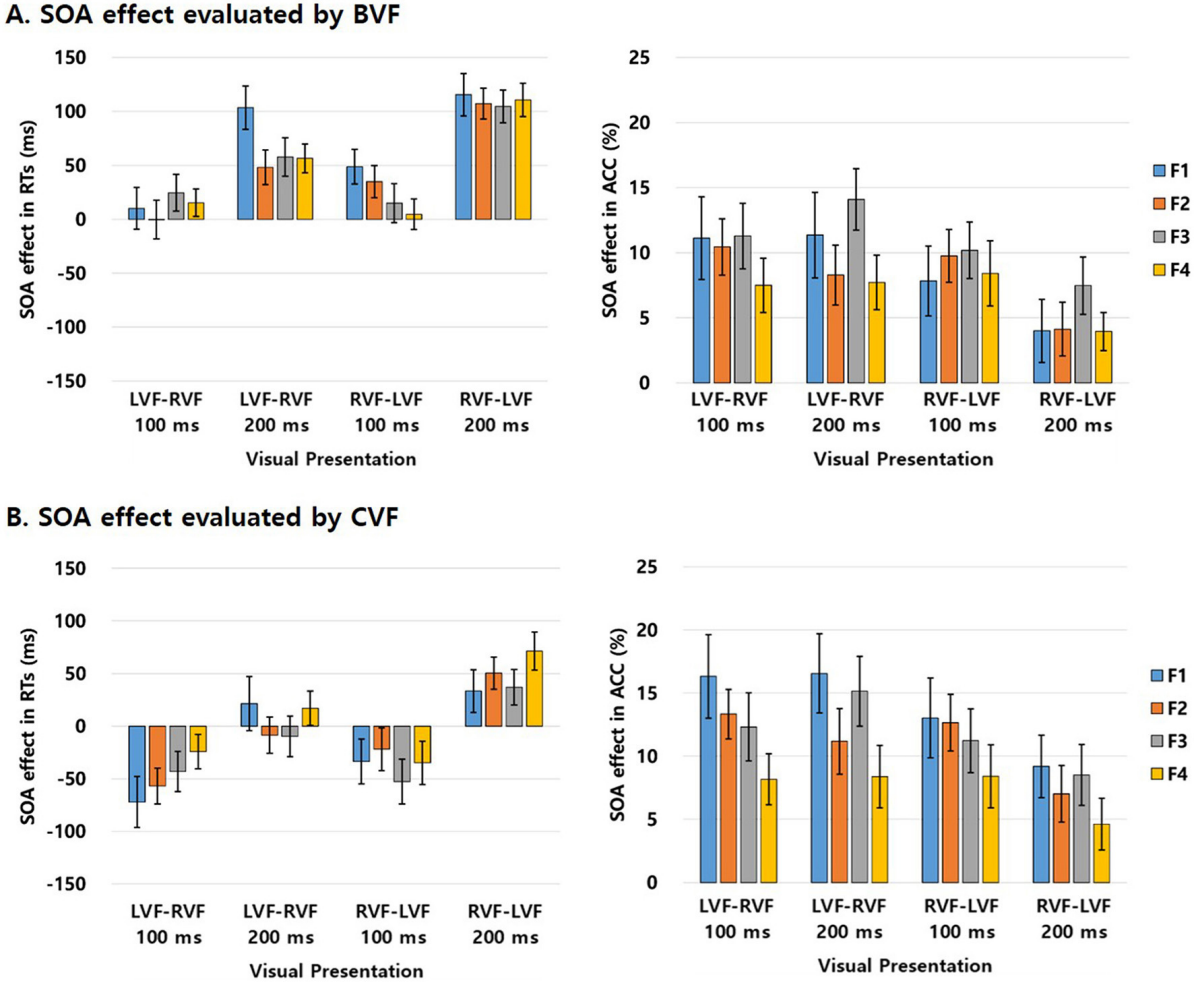
Graphical representation illustrating the comprehensive depiction of the extent of the SOA effect of words evaluated via BVF and CVF (SOA 0 ms), encompassing both RTs and ACC within each experimental condition, in relation to the familiarity effect in Experiment. **(A)** Magnitude of the SOA effect assessed through BVF. **(B)** Magnitude of the SOA effect assessed through CVF. The bars are accompanied by lines indicating standard errors. F1, The most unfamiliar level; F2, Slightly unfamiliar level; F3, Slightly familiar level; F4, The most familiar level.

Explored in detail within Table 4 is a comprehensive summary of the magnitude of the SOA effect evaluated by BVF in both RTs and ACC within each experimental condition for the familiarity effect. Additional visual representation of these findings is depicted in Figure 3. The SOA effect in bilateral presentation was evaluated for each subject to investigate whether SOA 0 ms significantly modulates the interhemispheric interactions on word processing between SOA 100 and 200 ms. Responses (RTs and ACC) of each VF and SOA condition were subtracted from the responses of the BVF (SOA 0 ms), and this value was defined as the SOA effect. Firstly, as we targeted on the effect of each participant, a three-way repeated measure analysis of variances (rm-ANOVAs) was performed, rather than a linear mixed-effects regression model, given that the latter is primarily suited for assessing the effects of independent variables on a single dependent variable rather than for comparing priming effects across multiple conditions (Kim et al., 2023b). So, the three-way rm-ANOVAs was conducted on the SOA effect in RTs, with factors of familiarity (F1/F2/F3/F4), VF (LVF-RVF/RVF-LVF), and SOA (100 ms/200 ms). The analysis revealed significant main effects of VF [*F*_(1, 40)_ = 10.840, *p* = 0.002, $\eta_{p}^{2}$ = 0.018] and SOA [*F*_(1, 40)_ = 129.275, *p* < 0.001, $\eta_{p}^{2}$ = 0.100], and a significant three-way interaction among all factors [*F*_(3, 120)_ = 2.749, *p* = 0.046, $\eta_{p}^{2}$ = 0.007]. Other effects, including the main effect of familiarity [*F*_(3, 120)_ = 1.035, *p* = 0.380, $\eta_{p}^{2}$ = 0.008] and two-way interactions between all factors [*F*_(1, 40)_ = 3.146, *p* = 0.084, $\eta_{p}^{2}$ = 0.005 for VF × SOA; *F*_(3, 120)_ = 0.906, *p* = 0.441, $\eta_{p}^{2}\;=$ 0.003 for familiarity × VF; *F*_(3, 120)_ = 0.650, *p* = 0.585, $\eta_{p}^{2}\;=$ 0.002 for familiarity × SOA], were not significant. The main effect of VF indicated a higher SOA effect in RVF-LVF presentation compared to LVF-RVF presentation, and the main effect of SOA revealed a higher SOA effect in SOA 200 ms compared to SOA 100 ms. The three-way interaction indicated significant simple two-way interactions between familiarity and SOA for both LVF-RVF [*F*_(1, 320)_ = 20.303, *p* < 0.001, $\eta_{p}^{2}$ = 0.058] and RVF-LVF presentations [*F*_(1, 320)_ = 54.037, *p* < 0.001, $\eta_{p}^{2}$ = 0.143]. However, no significant simple main effects of familiarity were observed in any of the conditions [LVF-RVF with SOA 100 ms: *F*_(3, 160)_ = 0.369, *p* = 0.775, $\eta_{p}^{2}$ = 0.007; LVF-RVF with SOA 200 ms: *F*_(3, 160)_ = 2.175,*p* = 0.093, $\eta_{p}^{2}$ = 0.039; RVF-LVF with SOA 100 ms: *F*_(3, 160)_ = 1.554, *p* = 0.203, $\eta_{p}^{2}$ = 0.028; RVF-LVF with SOA 200 ms: *F*_(3, 160)_ = 0.084,*p* = 0.969, $\eta_{p}^{2}$ = 0.002].

**Table 4:** Comprehensive summary of the magnitude of the SOA effect assessed through BVF and CVF (SOA 0 ms) in both RTs (ms) and ACC (%) within each experimental condition for the interhemispheric familiarity effect.

**Criterion**	**VF**	**SOA (ms)**	**Level of familiarity**							
			**F1**		**F2**		**F3**		**F4**	
			**RTs**	**ACC**	**RTs**	**ACC**	**RTs**	**ACC**	**RTs**	**ACC**
BVF	LVF-RVF	100	10 (124)	11.1 (20.3)	0 (110)	10.4 (16.2)	25 (115)	11.3 (13.9)	15 (82)	7.5 (13.4)
		200	103 (128)	11.4 (21.0)	48 (114)	8.3 (15.1)	58 (103)	14.1 (14.7)	56 (85)	7.7 (13.4)
	RVF-LVF	100	49 (103)	07.8 (17.2)	35 (116)	9.8 (13.9)	15 (95)	10.2 (13.0)	5 (91)	8.4 (16.0)
		200	116 (126)	8.4 (16.0)	107 (98)	4.1 (14.2)	105 (92)	4.0 (15.5)	111 (99)	3.9 (9.3)
CVF	LVF-RVF	100	−72 (155)	16.3 (21.1)	−57 (122)	13.3 (17.2)	−43 (108)	12.3 (12.5)	−24 (104)	0.8.2 (0.12.9)
		200	21 (165)	16.6 (20.1)	−9 (124)	11.2 (17.7)	−10 (110)	79.5 (17.2)	17 (104)	0.8.4 (0.15.8)
	RVF-LVF	100	−33 (137)	13.0 (20.2)	−22 (136)	12.7 (16.2)	−53 (130)	11.2 (14.4)	−35 (132)	0.8.4 (0.16.0)
		200	33 (130)	9.2 (15.8)	50 (108)	7.0 (15.5)	37 (98)	8.5 (14.4)	71 (116)	0.4.6 (0.13.1)

The values enclosed in brackets indicate the corresponding standard deviations.

Secondly, a three-way rm-ANOVAs was conducted on the SOA effect in ACC, with factors of familiarity (F1/F2/F3/F4), visual field (VF; LVF-RVF/RVF-LVF), and SOA (100 ms/200 ms). The analysis revealed a significant main effect of SOA [*F*_(1, 40)_ = 4.566, *p* = 0.969, $\eta_{p}^{2}$ = 0.039] and a significant two-way interaction between VF and SOA [*F*_(1, 40)_ = 6.853, *p* = 0.969, $\eta_{p}^{2}$ = 0.012]. Other effects, including the main effects of VF [*F*_(1, 40)_ = 4.008, *p* = 0.052, $\eta_{p}^{2}$ = 0.012] and familiarity [*F*_(3, 120)_ = 0.949, *p* = 0.419, $\eta_{p}^{2}$ = 0.009], two-way interactions between familiarity and VF [*F*_(3, 120)_ = 1.230, *p* = 0.302, $\eta_{p}^{2}$ = 0.002] and familiarity and SOA [*F*_(3, 120)_ = 0.832, *p* = 0.479, $\eta_{p}^{2}$ = 0.002], and the three-way interaction among all factors [*F*_(3, 120)_ = 0.062, *p* = 0.980, $\eta_{p}^{2}$ = 0.001], were not significant. The main effect of SOA indicated a higher SOA effect in SOA 100 ms compared to SOA 200 ms. Further analysis of the significant two-way interaction revealed that the simple main effect of SOA was not significant in LVF-RVF presentation [*F*_(1, 326)_ = 0.025, *p* = 0.875, $\eta_{p}^{2}$ = 0.001], whereas it was significant in RVF-LVF presentation [*F*_(1, 326)_ = 7.108, *p* = 0.008, $\eta_{p}^{2}$ = 0.021].

### Impact of SOA 0 ms on interhemispheric interactions assessed through CVF performance

The SOA effect assessed through CVF in terms of both RTs and ACC within each experimental condition for the familiarity effect was shown in Table 4. Further graphical representation of these outcomes can be observed in Figure 3. The SOA effect in foveal presentation was assessed for each subject to investigate whether an SOA of 0 ms significantly modulates the interhemispheric interactions on word processing between SOA 100 and 200 ms. Responses (RTs and ACC) for each VF and SOA condition were subtracted from the responses of the CVF (SOA 0 ms), aligning with the situation where the SOA is 0 ms and foveal stimuli are presented to both hemispheres simultaneously. This value was termed the SOA effect. Firstly, a three-way rm-ANOVAs was conducted on the SOA effect in RTs, with factors of familiarity (F1/F2/F3/F4), VF (LVF-RVF/RVF-LVF), and SOA (100 ms/200 ms). The analysis revealed significant main effects of VF [*F*_(1, 40)_ = 10.840, *p* = 0.002, $\eta_{p}^{2}$ = 0.013] and SOA [*F*_(1, 40)_ = 129.275, *p* < 0.001, $\eta_{p}^{2}$ = 0.072], and a significant three-way interaction among all factors [*F*_(3, 120)_ = 2.749, *p* = 0.046, $\eta_{p}^{2}$ = 0.005]. The main effect of familiarity was not significant [*F*_(3, 120)_ = 0.915, *p* = 0.436, $\eta_{p}^{2}$ = 0.006], nor were the two-way interactions between VF and SOA [*F*_(1, 40)_ = 3.146, *p* = 0.084, $\eta_{p}^{2}$ = 0.004], familiarity and VF [*F*_(3, 120)_ = 0.906, *p* = 0.441, $\eta_{p}^{2}$ = 0.002], and familiarity and SOA [*F*_(3, 120)_ = 0.650, *p* = 0.585, $\eta_{p}^{2}$ = 0.001]. The significant main effects indicated a higher SOA effect in RVF-LVF presentation than LVF-RVF, and a higher SOA effect in SOA 200 ms than SOA 100 ms. Further analysis of the significant three-way interaction revealed simple main effects of SOA in both LVF-RVF [*F*_(1, 320)_ = 15.100, *p* < 0.001, $\eta_{p}^{2}$ = 0.044] and RVF-LVF presentations [*F*_(1, 320)_ = 37.344, *p* < 0.001, $\eta_{p}^{2}$ = 0.103], indicating higher SOA effects in SOA 200 ms than SOA 100 ms. Simple main effects of familiarity and simple two-way interactions between familiarity and SOA were not significant in either presentation condition [*F*_(3, 320)_ = 0.839, *p* = 0.474, $\eta_{p}^{2}$ = 0.007 for familiarity; *F*_(3, 320)_ = 0.935, *p* = 0.424, $\eta_{p}^{2}$ = 0.008 for familiarity × SOA in LVF-RVF; *F*_(3, 320)_ = 0.794, *p* = 0.498, $\eta_{p}^{2}$ = 0.007 for familiarity; *F*_(3, 320)_ = 0.423, *p* = 0.736, $\eta_{p}^{2}$ = 0.004 for familiarity × SOA in RVF-LVF].

A subsequent analysis was conducted to evaluate the SOA effect in ACC, employing a three-way rm-ANOVA with factors of familiarity (F1/F2/F3/F4), visual field (VF: LVF-RVF/RVF-LVF), and SOA (SOA: 100 ms/200 ms). The analysis yielded significant main effects for all factors: familiarity [*F*_(3, 120)_ = 43.072, *p* < 0.001, $\eta_{p}^{2}$ = 0.186], VF [*F*_(1, 40)_ = 64.195, *p* < 0.001, $\eta_{p}^{2}$ = 0.461], and SOA [*F*_(1, 40)_ = 50.124, *p* < 0.001, $\eta_{p}^{2}$ = 0.035]. Additionally, significant two-way and three-way interaction effects were observed: familiarity × VF [*F*_(3, 120)_ = 78.112, *p* < 0.001, $\eta_{p}^{2}$ = 0.162], familiarity × SOA [*F*_(3, 120)_ = 66.799, *p* < 0.001, $\eta_{p}^{2}$ = 0.177], and familiarity × VF × SOA [*F*_(3, 120)_ = 67.235, *p* < 0.001, $\eta_{p}^{2}$ = 0.162]. The main effects revealed the largest SOA effect in F2, with similar magnitudes between F1 and F3, and the smallest effect in F4. The VF main effect indicated a higher SOA effect in LVF-RVF presentation compared to RVF-LVF, and the SOA main effect showed a higher effect in SOA 200 ms than SOA 100 ms. Further analysis of the significant three-way interaction revealed significant simple main effects of familiarity and SOA [*F*_(3, 320)_ = 82.500, *p* < 0.001, $\eta_{p}^{2}$ = 0.280 for familiarity; *F*_(1, 320)_ = 75.45, *p* < 0.001, $\eta_{p}^{2}$ = 0.085 for SOA] and a significant simple two-way interaction between familiarity and SOA in LVF-RVF presentation [*F*_(3, 320)_ = 80.840, *p* < 0.001, $\eta_{p}^{2}$ = 0.274]. This indicated the highest SOA effect in F2 and a higher effect in SOA 200 ms than SOA 100 ms for LVF-RVF presentation. Further analysis showed a significant simple main effect of familiarity in LVF-RVF presentation with SOA 200 ms [*F*_(3, 160)_ = 149.200, *p* < 0.001, $\eta_{p}^{2}$ = 0.737]. In RVF-LVF presentation, the simple main effect of SOA was significant [*F*_(1, 320)_ = 5.222, *p* = 0.023, $\eta_{p}^{2}$ = 0.016], indicating a higher SOA effect in SOA 100 ms than SOA 200 ms. However, the simple main effect of familiarity [*F*_(3, 320)_ = 1.273, *p* = 0.284, $\eta_{p}^{2}$ = 0.012] and the simple two-way interaction effect [*F*_(3, 320)_ = 0.119, *p* = 0.949, $\eta_{p}^{2}$ = 0.001] were not significant in this presentation condition.

## Discussion

In this study, we aimed to investigate the alterations in interhemispheric interactions during the development of reading proficiency, using word familiarity as an indicator of processing efficiency. Specifically, we sought to examine how the SOA between prime and target presentations modulates interhemispheric interaction during visual word processing, with a particular focus on the familiarity effect. To achieve this, we employed two SOA intervals: 100 and 200 ms. Additionally, two control conditions were incorporated, featuring an SOA of 0 ms, which included both BVF and CVF presentations. These control conditions were designed to examine the characteristics of the interhemispheric familiarity effect.

### Changes in interhemispheric interactions according to word familiarity

The findings revealed a significant interhemispheric familiarity effect in the sequential presentation involving the LVF/RH-RVF/LH prime and target presentation with an SOA of 100 ms, and the RVF/LH-LVF/RH prime and target presentation with an SOA of 200 ms. The significant familiarity effect in the LVF/RH-RVF/LH with the SOA of 100 ms aligns with the RH's specialization in visual-perceptual processing. Conversely, the significant familiarity effect in the RVF/LH-LVF/RH prime and target presentation with the SOA of 200 ms suggests that primary lexical processing related to word familiarity processing in the LH commences at a later stage following visual-perceptual processing. This explains the absence of a significant familiarity effect in the RVF/LH-LVF/RH prime and target presentation with the SOA of 100 ms, as this duration might be insufficient for processing the visual word based on the lexical information of the LH. These asymmetric familiarity effects between the visual fields, according to SOA, imply an asymmetric strategy between the two hemispheres in visual word processing. This strategy appears to be involved in distinct phases of interhemispheric interaction for the development of proficiency in visual word processing. The specialization in visual-perceptual processing at the RH may correlate with early interhemispheric interaction for proficient word recognition, while the specialization in lexical processing at the LH may relate to interhemispheric interaction subsequent to the processing of visual-perceptual attributes. Therefore, the observation that the two hemispheres may engage in the proficiency of visual word processing at different timings (100 ms at the RH, 200 ms at the LH) and in a specific activated order (LVF/RH-RVF/LH, RVF/LH-LVF/RH) for the familiarity effect suggests that the nature of interhemispheric interactions may be dictated by the specialization of each hemisphere in visual word processing.

In addition, the analysis revealed a specific interplay between lexicality, VF, and SOA in ACC, contrasting with the findings in RTs. The three-way interaction effect manifested as enhanced ACC for visual recognition of non-words compared to words in the SOA 100 ms when the prime and target were sequentially presented at the LVF/RH and RVF/LH. Conversely, greater ACC for non-words was observed in both SOA of 100 and 200 ms when the prime and target were sequentially presented at the RVF/LH and LVF/RH. Interestingly, the lexicality effect appears to mirror the findings of the familiarity effect, as it was evident in the LVF/RH-RVF/LH prime and target presentation with the SOA 100 ms and in the RVF-LVF prime and target presentation with the SOA 200 ms. However, a distinction emerged between the lexicality and familiarity effects, as the former demonstrated a significant effect in the RVF/LH-LVF/RH prime and target presentation with the SOA 100 ms. The comparison of performances within the familiarity condition for words, known as the familiarity effect, differs slightly from the comparison of performances between words and non-words, termed the lexicality effect. This distinction arises because the lexical decision to discriminate between words and non-words may primarily necessitate visual-perceptual oriented processing, considering the orthographic differences in visual attributes that distinguish words from non-words. Consequently, the LH might leverage visual-perceptual information to discern whether stimuli are words or non-words. This interpretation aligns with previous studies (e.g., Blanca and López-Montiel, 2009; Boles, 1984; Lamb et al., 1989; Van Kleeck, 1989), which have emphasized the LH's superiority over the RH in processing the local features of visual stimuli. Research into hemispheric asymmetry for processing global versus local features of visual stimuli, conducted through visual half-field studies with neurologically intact individuals, has documented a LVF/RH advantage for identifying larger letters and a RVF/LH advantage for identifying smaller letters (Boles, 1984; Van Kleeck, 1989). Van Kleeck (1989) posited that a global/local interaction exists in neurologically intact individuals, predicated on visual field differences. Collectively, these findings underscore a hemispheric asymmetry in the global and local processing of visual stimuli, with global processing predominantly linked to the RH and local processing associated with the LH, meaning the potential existence of relatively weak visual-perceptual processing at the LH.

Thus, the observed lexicality effect, characterized by more accurate responses for non-words than for words, may be attributed to the LH's processing of local aspects of visual stimuli. This processing aligns with the requirements of lexical decision-making, which necessitates processing based on visual-perceptual attributes. Such an interpretation can investigate the significant lexicality effect in the RVF/LH-LVF/RH prime and target presentation, even when the SOA between the prime and target was only 100 ms. This brief interval seems adequate for the LH to engage in visual-perceptual processing for the lexical decision of non-words, even though this processing is relatively weaker in the LH compared to the RH. Contrastingly, the LH's visual-perceptual processing may be less adept at handling familiar words, as evidenced by the absence of a familiarity effect in the RVF/LH-LVF/RH prime and target presentation with the SOA of 100 ms. While the lexical decision for non-words may be achievable using visual-perceptual information, the lexical decision for words may demand processing grounded in other information, such as lexical attributes. This distinction could result in the observed lack of a familiarity effect in the RVF/LH-LVF/RH prime and target presentation with the SOA of 100 ms. In conclusion, the asymmetric familiarity and lexicality effects according to the VFs and SOAs may stem from the distinct processing strategies employed by each hemisphere. These findings lend support to the results of the familiarity effect and contribute to our understanding of the intricate interplay between hemispheric specialization, visual-perceptual processing, and lexical decision-making in visual word recognition.

### Modulation of interhemispheric interactions through the manipulation of SOA between bilaterally activated time intervals

The current study incorporated two control conditions, BVF and CVF, representing a SOA 0 ms, to investigate the SOA effect. This was achieved by subtracting the responses at SOA 100 and 200 ms from those at SOA 0 ms. We initially hypothesized the familiarity effect will not be found in the SOA effect since the bilateral activation significantly neutralizes the interhemispheric familiarity effect. Indeed, the results did not reveal a significant familiarity effect in any combination of experimental conditions between the VF and SOA in the SOA effect, as measured by both RTs and ACC in the BVF. Furthermore, the SOA effect analysis on RTs did not yield a significant familiarity effect across any experimental conditions between the VF and SOA. The highest SOA effect was observed at the F2 level in the familiarity condition, indicating non-linearity with the increases in word familiarity. The results of the familiarity effect, evaluated through the SOA effect employing both BVF and CVF in RTs and ACC, indicate that the simultaneous activation of both hemispheres is not significantly different with the pattern of interhemispheric interactions in the responses at SOA 100 and 200 ms according to the VFs. This observation underscores a significant modulation of SOA on the interhemispheric interaction within the 100 to 200 ms range, in alignment with the findings in the familiarity and lexicality effects in RTs and ACC.

The dual-route cascaded (DRC) model of visual word recognition offers a theoretical framework that posits two distinct processing pathways for word recognition: the “direct” and “indirect” routes. Developed to explain how skilled readers recognize words rapidly and accurately (Coltheart et al., 2001), the model suggests that familiar words are processed via direct access to stored representations in long-term memory, while less familiar words rely on a more sequential, sublexical processing route. The direct route involves holistic recognition, where the word is accessed as a whole, without the need for decomposition into individual components. In contrast, the indirect route involves phonological decoding, where individual graphemes or letters are processed to derive phonological representations.

The findings of the current study are consistent with the DRC model, particularly in terms of how interhemispheric interactions may vary depending on word familiarity. Words that are highly familiar are likely processed primarily by the LH through the direct route, where lexical processing relies on access to stored mental representations. The RH may then play a supporting role, particularly in processing visual features and integrating contextual information. Conversely, for less familiar words, the LH may engage more in sublexical processing via the indirect route, with the RH contributing to visual processing and contextual integration. In this explanation, the relative contributions of lexical and sublexical processing mechanisms in the LH and RH may shift, resulting in different patterns of interhemispheric interaction.

Therefore, the results of the present investigation indicate that interhemispheric interaction may manifest differently depending on the specific type of processing involved, namely visual-perceptual or lexical processing. This distinction arises from the differential specialization between the two hemispheres, with the RH primarily engaged in visual-perceptual processing and the LH in lexical processing. Consequently, the pattern of interhemispheric interaction may vary, demonstrating potential interaction from the RH to LH during visual-perceptual processing and from the LH to RH during lexical processing. In support of this notion, Kim et al. (2023a) employed a masked repetition priming paradigm for lateralized lexical decision and observed a stronger interhemispheric interaction for words from the LH to RH than from the RH to LH at SOAs of 120 and 600 ms. This finding suggests that the LH may be the primary hemisphere in visual word processing within these SOA ranges, aligning with the results observed at the SOA 200 ms in the current study. Such outcomes hint at the possibility of differential interhemispheric interaction, such as the interaction from the RH to LH at the SOA 100 ms. Furthermore, Kim et al. (2023b) reported variations in interhemispheric interactions depending on the temporal processing period in visual recognition of words as a function of visual familiarity. Utilizing Granger causality analysis during foveal word recognition, coupled with electrophysiological recording, they detected significant changes in interhemispheric interaction from the LH to RH and from the RH to LH in the N100 time range (130–210 ms), as well as a significant shift in interaction from the LH to RH in the N400 time range (400–500 ms). Thus, the findings of the current study suggest the existence of differential interhemispheric interactions at millisecond intervals, potentially attributable to asymmetric specialization between the two hemispheres in visual word processing.

Despite offering valuable insights into interhemispheric interaction during visual word recognition via repetition priming, our study acknowledges certain limitations. A primary limitation lies in our control conditions, specifically the 0 ms SOA conditions encompassing both CVF and BVF presentations. While these served as crucial baselines for examining SOA effects, the CVF condition inherently involves focused foveal attention, whereas the BVF condition necessitates divided parafoveal attention. This disparity in attentional allocation and visual acuity could introduce confounding factors that might influence our observed results. Therefore, future research should explicitly investigate the impact of attentional allocation and visual acuity on parafoveal lexical decision tasks and their subsequent effects on interhemispheric interactions in visual word recognition. Secondly, the current study relied exclusively on behavioral indicators, such as RTs and ACC. While these behavioral markers provided valuable insights, the absence of neuroscientific indicators from methods like electroencephalography (EEG) or functional magnetic resonance imaging (fMRI) limits our ability to infer direct neural mechanisms. Neuroscientific approaches offer superior temporal and spatial resolution, allowing for a more precise understanding of the causal relationships underlying the observed interhemispheric temporal and spatial dynamics. Consequently, future research employing neuroscientific methodologies is required to further examine these temporal and spatial dynamics. Finally, individual variations in cognitive ability may modulate lexical decision performance. For instance, Kim et al. (2025) explored how risk-taking propensity influences lexical decision tasks in neurologically intact participants. They reported that individuals with a higher propensity for risk-taking exhibited slower and less accurate word recognition, accompanied by a response bias toward both non-words and words. Importantly, despite these differences in overall performance, both high and low risk-taking groups demonstrated comparable patterns of lexical variable effects on word recognition. This suggests that risk-taking propensity does not introduce qualitative disparities in the underlying visual word recognition processes. These findings underscore that an individual's risk-taking level can indeed impact lexical decision outcomes, indicating the necessity of controlling for this variable in future research. Consequently, subsequent investigations should aim to elucidate how the time interval effect (SOA effect) might be modulated by such cognitive functions, including risk-taking propensity.

## Conclusion

The present study employed word familiarity to investigate the modulation of SOA between prime and target presentations on interhemispheric interactions during visual word recognition. Significant effects of familiarity for words were observed when the LVF prime and the RVF target were sequentially presented at an SOA of 100 ms, replicating the findings of Kim and Nam (2024) and highlighting the dominance of the RH's visual-perceptual specialization in interhemispheric interactions at this temporal interval. At an SOA of 200 ms, a significant familiarity effect emerged when an RVF prime and LVF target were presented, suggesting that the LH's primarily lexical processing exerts a stronger influence on interhemispheric interactions in visual word recognition at this longer SOA. Furthermore, no interhemispheric familiarity effect was observed when responses at the two SOA levels were compared to the SOA 0 ms control conditions, which involved BVF and CVF stimuli designed to activate both hemispheres simultaneously. This lack of familiarity effect suggests that simultaneous bilateral activation may attenuate the interhemispheric interactions seen in sequential presentations, likely because the interhemispheric familiarity effect in sequential conditions is consistent with that observed in simultaneous bilateral activation. In sum, this study demonstrates that the modulation of SOA between prime and target presentations influences visual word recognition, indicating that the impact of hemispheric specialization on visual word processing is dependent on the temporal interval between activations in the two hemispheres.

## Data Availability

The raw data supporting the conclusions of this article will be made available by the authors, without undue reservation.
